# Supplementary material for: Psycho-Behavioural Segmentation in Food and Nutrition: A Systematic Scoping Review of the Literature
Source: Nutrients. 2021 May 25;13(6):1795. doi: 10.3390/nu13061795 (PMC8226652; doi:10.3390/nu13061795)
Supplement: Supplementary file 1 [file nutrients-13-01795-s001.zip › Supplementary file 1.pdf]

Supplementary file 1. The different segmentation bases

Table 1. The different segmentation bases.

Segmentation base	Description	Examples	Example in practice	Method	Inferences
Demographic [1]	Creating groups on the basis of physical and factual data relating to populations and sub-groups within populations	Age, income, occupation, gender, religion, ethnicity, household structure, family size.	Targeting 18-24-year-old people living at home with their parents can be determined in advance.	A-priori	Social class, social status
Psychographic [2]	A method used to group prospective, current or previous customers by their shared psychological traits, values, motives, or interests.	Attitudes, opinions, beliefs, personality, values, interests, motivations, emotions, traits, cultural beliefs, subculture, Generational (e.g. Boomers), propensities (e.g. willingness to behave in certain ways).	Targeting young LGBTQI+ people and further segmenting them by their attitudes towards nutrition and exercise.	Hybrid	Future feelings and thoughts
Behavioural [3]	The grouping of people in a 'market' into homogeneous groups based on their mutual behavioural patterns.	Lifestyle, shopping choices, activities, hobbies, habits, loyalty, product or service usage, benefits sought, readiness to buy, stage of change, adoption of idea, media usage, viewing habits, social media usage, experiences, occasions of use, satisfaction levels, journey stage (e.g. beginning or ending).	In observing people in a shopping centre food court, we can see that certain people do not clear their tables after use. Surveying additional food related behaviours can create meaningful segments for future campaigns.	Hybrid	Future behaviours
Psycho-behavioural	Combines psychographic and behavioural variables to create groups based on what people do and why they behave the way they do.	Motivation to eat, beliefs about how to maintain a healthy diet, attitude towards participating in physical activity.	Targeting young adults based on their attitude towards healthy eating and their dietary-related behaviours.	Hybrid	Future feelings, thoughts, and behaviours
Geographic [1]	Forming groups of people (i.e. the market) on the basis of where they are located.	Home address, work or study address, destinations (e.g., travel, leisure), countries, regions, cities, states, regional/rural, urban/suburban, weather patterns, climate.	Targeting people from metropolitan Melbourne.	A-priori	Social class, social status

References

1. Camilleri MA. Market Segmentation, Targeting and Positioning. In: Travel Marketing, Tourism Economics and the Airline Product: An Introduction to Theory and Practice. Cham: Springer International Publishing; 2018. p. 69–83. (Tourism, Hospitality & Event Management).

2. Hardcastle SJ, Hagger MS. Psychographic Profiling for Effective Health Behavior Change Interventions. Front Psychol. 2015;6:1988.

3. Kitunen A, Rundle-Thiele S, Kadir M, Badejo A, Zdanowicz G, Price M. Learning what our target audiences think and do: extending segmentation to all four bases. BMC Public Health. 2019 Apr 5;19(1):382.
