# Supplementary material for: Psycho-Behavioural Segmentation in Food and Nutrition: A Systematic Scoping Review of the Literature
Source: Nutrients. 2021 May 25;13(6):1795. doi: 10.3390/nu13061795 (PMC8226652; doi:10.3390/nu13061795)
Supplement: Supplementary file 1 [file nutrients-13-01795-s001.zip › Supplementary File 2.pdf]

Supplementary File 2: Full search strategy detailing the database searches conducted on 30th June 2020

OVID Medline search: 2,696 results

- Limit to 2010
- **Map to subject headings:** cluster analysis/ or latent class analysis/ OR
    - segment* or "Cluster analys*" or clustering* or "Latent class analys*" or "K-means*" or kmeans* or "peer crowd*" or "principal component analys*" or "discriminant analys*"
- **Map to subject headings:** Feeding behaviour OR diet, healthy OR food fussiness/preferences OR
    - ((health* or unhealth* or habit*) adj2 (eat* or food* or diet or diets or behav*))

PsycINFO: 835 results

- Limit to 2010
- Map to subject headings: cluster analysis/ or latent class analysis/ OR
    - segment* or "Cluster analys*" or clustering* or "Latent class analys*" or "K-means*" or kmeans* or "peer crowd*" or "principal component analys*" or "discriminant analys*"
- Map to subject headings: Feeding behaviour OR diet, healthy OR food fussiness/preferences OR
    - ((health* or unhealth* or habit*) adj2 (eat* or food* or diet or diets or behav*))

Scopus: 2,770 results

- Limit to 2010 and journal articles only

(TITLE-ABS-KEY (segment* OR "Cluster analys*" OR clustering* OR "Latent class analys*" OR "K-means*" OR kmeans* OR "peer crowd*" OR "principal component analys*" OR "discriminant analys*") AND TITLE-ABS-KEY ("health* eat*" OR "eat* health*" OR "eat* unhealth*" OR "unhealth* eat*" OR "health* food*" OR "unhealth* food*" OR "health* diet" OR "unhealth* diet" OR "health* diets" OR "unhealth* diets" OR "eat* habit*" OR "health* behav*" OR "unhealth* behav*" OR "food habit*")) AND (LIMIT-TO (DOCTYPE , "ar")) AND (LIMIT-TO (PUBYEAR , 2020) OR LIMIT-TO (PUBYEAR , 2019) OR LIMIT-TO (PUBYEAR , 2018) OR LIMIT-TO (PUBYEAR , 2017) OR LIMIT-TO (PUBYEAR , 2016) OR LIMIT-TO (PUBYEAR , 2015) OR LIMIT-TO (PUBYEAR , 2014) OR LIMIT-TO (PUBYEAR , 2013) OR LIMIT-TO (PUBYEAR , 2012) OR LIMIT-TO (PUBYEAR , 2011) OR LIMIT-TO (PUBYEAR , 2010)) AND (LIMIT-TO (LANGUAGE , "English"))

CINAHL Plus: 1,495 results

- Limit to 2010 and journal articles

("health* eat*" OR "eat* health*" OR "eat* unhealth*" OR "unhealth* eat*" OR "health* food*" OR "unhealth* food*" OR "health* diet" OR "unhealth* diet" OR "health* diets" OR "unhealth* diets" OR "eat* habit*" OR "health* behav*" OR "unhealth* behav*" OR "food habit*") AND (segment* OR "Cluster analys*" OR clustering* OR "Latent class analys*" OR "K-means*" OR kmeans* OR "peer crowd*" OR "principal component analys*" OR "discriminant analys*")

Business Source Complete: 124 results

- Limit to 2010 and journal articles

("health* eat*" OR "eat* health*" OR "eat* unhealth*" OR "unhealth* eat*" OR "health* food*" OR "unhealth* food*" OR "health* diet" OR "unhealth* diet" OR "health* diets" OR "unhealth* diets" OR "eat* habit*" OR "health* behav*" OR "unhealth* behav*" OR "food habit*") AND (segment* OR "Cluster analys*" OR clustering* OR "Latent class analys*" OR "K-means*" OR kmeans* OR "peer crowd*" OR "principal component analys*" OR "discriminant analys*")

Emerald Insight: 1,556 results

- Limit to 2010 onwards and articles only

(segment* OR "Cluster analys*" OR clustering* OR "Latent class analys*" OR "K-means*" OR kmeans* OR "peer crowd*" OR "principal component analys*" OR "discriminant analys*") AND ("health* eat*" OR "eat* health*" OR "eat* unhealth*" OR "unhealth* eat*" OR "health* food*" OR "unhealth* food*" OR "health* diet" OR "unhealth* diet" OR "health* diets" OR "unhealth* diets" OR "eat* habit*" OR "health* behav*" OR "unhealth* behav*" OR "food habit*")
