# Supplementary material for: Psycho-Behavioural Segmentation in Food and Nutrition: A Systematic Scoping Review of the Literature
Source: Nutrients. 2021 May 25;13(6):1795. doi: 10.3390/nu13061795 (PMC8226652; doi:10.3390/nu13061795)
Supplement: Supplementary file 1 [file nutrients-13-01795-s001.zip › Supplementary File 4.pdf]

Supplementary File 4: Full version of Table 2 with key results and segment descriptions and demographics

Author; year; location	Underlying theory or models	n segmented; age (mean (SD) or otherwise specify); gender/sex[a]	Tool used for segmentation *Variables/question/items used*	Segmentation method	No. segments	Segment number, name (% of sample), demographics, and description
Brečić et al; 2017; Croatia	N/R	500; 47.6 (SD N/R); 46.8% male, 53.2% female	Food Choice Questionnaire (modified). *Questions under 4 factors (health and sensory, price, digestion, and convenience).*	Cluster analysis; Ward's method	4	**Segment 1: Healthy and tasty food lovers - 23.6%**<br>This segment is sensitive to intrinsic properties of food and worried about additives and artificial ingredients. They prefer food with vitamins/minerals which tastes/looks good and has environmentally friendly packaging. Food preparation, availability, price and country of origin are not important and they frequently eat all types of food. This group consists of 44% males and 56% females, they consider their health as good (62%) or moderate (30%). Two-thirds live with partners and they have the highest level of education of the sample.<br>**Segment 2: Convenient consumers - 26.9%**<br>This group mainly consume traditional and functional food. They are mostly concerned about extrinsic quality characteristics such as ease of preparation, price and availability. Natural content, composition, personal attachment and country of origin is less important. This group consists of 58% males and 42% females who are mostly single, mostly consider their health as good (63%) or moderate (32%), are under 40 years, and from urban areas.<br>**Segment 3: Concerned consumers - 27.2%**<br>This segment is concerned about both intrinsic and extrinsic characteristics. It is important for them that food has vitamins, minerals and no additives/artificial ingredients. They prefer food low in calories, with low fat and high fibre content. Food texture, availability, countries of origin, appearance, familiarity, value for money and influence of mood and health are important. They consume specialty foods less often. This group consists of 36% males and 64% females, nearly half consider their health as good (46%), and is the oldest segment (mean age 50yrs).<br>**Segment 4: Indifferent consumers - 19.9%**<br>Intrinsic and extrinsic characteristics are not important to this group, and

	they are the least worried about food additives, artificial ingredients, taste, price, nutritional value and impact on mood and health. They consume traditional and organic food more often than functional. This group consists of 53% males and 47% females.

Brennan et al; 2020; Australia	Transtheoretical model of behaviour change	195; 21.0 (2); 39.0% male, 61.0% female	Themes associated with dietary behaviours and attitudes towards eating.	Qualitative thematic analysis	3	**Segment 1: Saints**[f]<br>Saints are in agreement with the ideals and behaviours associated with healthy eating. They are also characterised by balance: they can eat discretionary foods without feeling guilty. They are self-determined and their rewards for their healthy eating behaviours are inherent, and therefore they are motivated to continue.<br>**Segment 2: Sinners**[f]<br>Sinners were those who opposed healthy eating ideals. They did not practice the 'religion' of healthy eating. Sinners indicated they were content with their unhealthy lifestyle choices.<br>**Segment 3: Person in the pew**[f]<br>The person in the pew was characterised by espoused beliefs in the ideals of healthy eating but an overall lack of 'active engagement' with the actions and behaviours required to perform healthy eating within their lifestyles. They did not plan to make any immediate changes to their diet, although they may consider doing so in the future. They sometimes/often had healthy eating knowledge and beliefs, and felt like they should be eating healthier, which led to feeling guilty.

Burton et al; 2017; Australia	Theory of planned behaviour	1059; >18-61+(range); 35.3% male, 64.7% female	Developed their own questionnaire based on scales previously used in the literature. *Questions from 2 scales: perceived cooking capability and perceived nutrition knowledge.*	Cluster analysis; 2 step	3	**Segment 1: Low confidence for nutrition knowledge and cooking capability - 22.9%**<br>This segment had the lowest scores for all six confidence items with a total mean perceived nutrition knowledge and confidence score of 2.96/7. This group is most likely to report impulse food purchasing. They are generally less educated (significantly more likely to have only completed high school) and have a higher BMI.<br>**Segment 2: Moderate confidence for nutrition knowledge and cooking capability - 48.3%**<br>This group has few barriers to healthy eating with a total mean nutrition knowledge and cooking confidence score of 4.43/7.<br>**Segment 3: High confidence for nutrition knowledge and cooking capability - 28.8%**<br>This segment has the highest positive attitudes towards food, healthy eating and eating intentions. They have more perceived behavioural control and higher overall diet and bodyweight satisfaction. They report less barriers to healthy eating with a total mean food knowledge and cooking confidence score of 6.00/7.They have the highest meal formality score and are most likely to use a shopping list and product information. They focus on fresh foods, have highest vegetable prominence in meals, use fewest convenience ingredients and have fewer time constraints in meal preparation. This group is generally more highly educated, are less likely to be in the low income category, and have a lower BMI (although still in the overweight range).<br>There were no significant differences between the three clusters for gender, age, or the presence of children under 18 years living at home.

Cabral et al; 2017; Cape Verde	N/R	Study 1: 433; 35.9 (6.4); 35.6% male[a], 64.4% female[a]<br><br>Study 2: 119; 35.2 (17.7); 34.6% male[a], 65.4% female[a]	Food Choice Questionnaire (Portugese version). *Questions under 9 factors (nutrition and diet, sensory appeal, mood, wellbeing, convenience, price, familiarity, ethical concern, natural content).*	Cluster analysis; 2 step	3	**Segment 1: Healthy - S1 12%, S2 25%**<br>This segment assigned higher ratings to the natural content in food, their wellbeing, and nutritional aspects of their diet. They gave the least importance to the price, familiarity and convenience of food. This group has a higher level of education, lives mostly in the city and has fewer children.<br>**Segment 2: Hedonists - S1 35.7%, S2 51%**<br>This segment gave the highest rating to the sensory appeal of food and assigned lower ratings to the natural content of food, their wellbeing, and the nutritional aspect of their diet. This group had the lowest education of all segments.<br>**Segment 3: Engaged - S1 52.3%, S2 24%**<br>This segment attributes high ratings to all factors. They appear to be more concerned with their daily choices and all the aspects of food choice. This segment was the oldest (mean age 36.6yrs).

den Uijl et al 2016; The Netherlands	N/R	Study 1: 392; 65.8 (5.9); 40.3% male[a], 59.7% female[a]<br><br>Study 2: 40; 66.9 (4.8); 50.0% male[a], 50.0% female[a]	Mealtime functionality questionnaire. *Questions under 13 constructs (hunger, habit, liking, cosiness, pleasure, energising, rewarding, healthiness, pleasing, calming, physical needs, thoughtless eating, and environmental awareness).*	Cluster analysis; hierarchical complete linkage	Study 1: 3; Study 2: 2	**Study 1:**<br>**Segment 1: Cosy socialisers - 28%**<br>This segment considers meals to be a cosy/sociable occasion. They eat because they like it and associate positive emotions with meals. This segment had an average age of 66.4 years and 43% of this group are male. They reported an excellent quality of life more frequently than the other clusters.<br>**Segment 2: Physical nutritioners - 39%**<br>This segment believes meals are an occasion to combat hunger and to get energy. They eat because it is healthy, pleasing and they associate mainly positive emotions with their mealtimes. This segment had an average age of 64.9 years, were 33% male, and reported an average quality of life.<br>**Segment 3: Thoughtless rewarders - 33%**<br>This segment eat without having explicit thoughts about it and are not concerned about health. They linked meal occasions more strongly to the negative emotions - guilty and disgusted. This segment had an average age of 66.4 years, and were 47% males.<br>**Study 2:**<br>**Segment 1: Cosy socialisers - 50%**[f]<br>This segment found evening meals provided cosiness/social interaction and were a time to relax. Social connectedness was achieved by eating together, serving a glass of wine and setting the table. They enjoy exploring new meals and products. They liked to increase their daily protein intake by eating more natural products.<br>**Segment 2: Physical nutritioners - 50%**[f]<br>This segment consumes evening meals to combat hunger and thirst and to feel satisfied. They eat a lot of vegetables because these contain important nutrients to fulfil physical requirements, make them feel good and stay fit and healthy. They dislike food waste and most prefer to increase their protein intake by adding protein powder to their diet.

Espinoza-Ortega et al; 2016; Mexico	N/R	202; 18-60 (range); 42.5% male[a], 57.2% female[a]	Food Choice Questionnaire (modified). *Questions under 10 factors (care for weight and health, social sensitivity, practicality, economic aspects, not industrialised, hedonism, traditionality, familiarity, no sugar).*	Cluster analysis; Ward's method	4	**Segment 1: Traditional - 20.1%**<br>This segment had the highest scores in eight (of ten) factors. They do not pay attention to weight and health or avoid sugar consumption. This segment has a lower education than others.<br>**Segment 2: Healthy not committed - 41.6%**<br>This segment pays attention to weight and health factors and avoids the consumption of foods with sugar. They are younger than other segments.<br>**Segment 3: Conscious - 27.0%**<br>This segment had the highest scores in all factors. They give more importance to weight and health care, and avoid consuming foods with sugar. They are most concerned with the care of the environment and animal welfare, and are more reflexive and sensitive not only to their surroundings but also with their own body.<br>**Segment 4: Careless - 11.3%**<br>This segment does not care about weight and health care and consuming foods with sugar (lowest scores). They are pragmatic consumers who give more importance to issues related to practicality, familiarity, traditionality, and economic aspects of food. They are younger than other segments and have a higher proportion of men.
Gama et al; 2018; Malawi	N/R	489; 18-50+ (range); 68% male, 32% female	Food Choice Questionnaire (modified). *Questions under 5 factors (mood, health, price and preparation convenience, sensory appeal, familiarity).*	Cluster analysis; Ward's method	4	**Segment 1[d] - 30.0%**<br>This segment's food choices were influenced by mood, health, price, preparation convenience, familiarity, and sensory appeal. This segment also had lower monthly incomes compared to others.<br>**Segment 2[d] - 13.0%**<br>This segment was indifferent to all factors affecting food choice. They were more likely to have a lower education level (high school and below).<br>**Segment 3[d] - 33.0%**<br>This group was concerned about familiarity and indifferent to health, price, and preparation convenience. They were more likely to have a lower education level (high school and below).<br>**Segment 4[d] - 24.0%**<br>This group was more concerned about health but indifferent to mood and familiarity. This segment had a higher monthly income, were younger, and men were 1.5 times more likely to be in this segment than women.

Grunert et al; 2011; China	N/R	479; 39 (1.8); 50.1% male, 49.9% female	Food-Related Lifestyle Instrument (modified). *Questions from 5 dimensions (ways of shopping, quality aspects, cooking methods, consumption situation, purchase motives).*	Latent class cluster analysis	3	**Segment 1: Concerned - 45.0%[e]**<br>This segment placed the largest importance on food quality aspects; particularly freshness, price/quality, and healthiness, ingredients, and placed the least importance on food taste and eating out. They consider social relationships as an important food purchasing motive. This segment placed small importance on planning, cooking and on other aspects such as enjoyment of shopping, involvement in food shopping and use of a shopping list. They are concerned about the environment and nature, and expressed stronger worries about food production and the environment. This segment has slightly more females (56%) than males.<br>**Segment 2: Uninvolved - 33.0%[e]**<br>This segment was generally neutral across most of the food-related lifestyle dimensions. They were the least concerned consumers of the whole sample about the environment and nature, and food production and the environment, with importance scores slightly higher than the neutral point. This segment is equal male and female consumers.<br>**Segment 3: Traditional - 21.0%[e]**<br>This segment placed importance on food quality aspects (particularly freshness) and placed emphasis on taste. They also placed the highest importance of all clusters on the contribution of the whole family to meal/eating preparation and certain food purchase motives such as social relationships and security. They assigned the least importance of all clusters to several aspects of shopping e.g. use of a shopping list, and to aspects of cooking e.g. planning. This segment had more males (64%) than females.

Gunden et al; 2020; Turkey	Theory of Reasoned Action; Theory of Planned Behaviour	371; N/R for total sample; 46% male, 54% female	Developed their own questionnaire based on scales previously used in the literature. *Questions from the green values scale - 6 statements about environmental beliefs.*	Factor analysis	2	**Segment 1: Positive perceivers - 62.3%**<br>This segment is more inclined to behave environmentally friendly. They have healthier and environmentally friendly food consumption behaviours compared to the negative perceivers. These consumers read food labels, buy ecological food products, eat fresh fish and fruit, eat with family, and spend a lot of time cooking and planning their meals. This segment is older (mean age 42yrs) and had a higher proportion of females (58%). A proportion of this segment smoked (37%) and only 25% exercised regularly.<br>**Segment 2: Negative perceivers - 37.7%**<br>This segment is less inclined to behave environmentally friendly. They are open to new taste experiences in food purchase, eat sweet desserts and snack food, eat fast food, drink alcohol, and use pre-packaged dishes rather than cooking from scratch. This segment is younger (mean age 35yrs), 53% male, and only 17% exercise regularly.
Keller et al.; 2019; Azerbaijan	N/R	419; 26.33 (3.18); 100% female[c]	Three Factor Eating Questionnaire (modified). *Questions under 3 factors (emotional eating, cognitive control, uncontrolled eating).*	Cluster analysis; Ward's method	3	**Segment 1: Functional eaters – 36.6%**<br>This segment agreed with the statements of uncontrolled eating. They are not familiar with any of the eating styles and are not influenced by weight control, emotions or external stimuli (eg. delicious food or other people eating). They have an average level of income (67.1%), hold a bachelor's degree (68.4%) and are typically single (78.9%). This group participates in moderate physical activity (65.1%) and have a normal weight based on their BMI (90.1%).<br>**Segment 2: Conscious eaters - 27.2%**<br>This segment disagrees with emotional eating and is not affected by external stimuli however, they care about eating and pay attention to their food choices. They are more likely to be characterised by cognitive restrained eating. This segment has a high level of income (78.9%) and majority hold a bachelor's (65.8%) or master's (34.2%) degree. They do regular physical activity 1-3 times/week and although all the underweight females belong to this group, they can be characterised by normal body weight (92.0%).<br>**Segment 3: Emotional and hedonic eaters - 36.5%**<br>This segment can be characterised by emotional and uncontrolled eating. They often feel hungry, always eat when they are hungry and able to eat at any time. They eat due to negative feelings, food is considered a 'bonus'

						and they are influenced by external stimuli. Majority of this group have a low-income level (66.7%), hold an associate (47.1%) or bachelor's degree (43.1%) and are single (62.7%). This group follows a sedentary lifestyle (86.3%); none of them do a high level of physical activity and more than two-thirds of them are overweight or obese (86.9%).
Kitunen et al; 2019; Australia	The Motivation, Opportunity, and Ability (MOA) theoretical framework	327; 20-35 (range)[b]; 22.9% male[c], 77.1% female[c]	Developed their own questionnaire based on scales previously used in the literature. *Questions about education level, motivation, ability, opportunity, and Turconi eating behaviour score.*	Cluster analysis; 2 step	2	**Segment 1: Breakfast skippers - 48.6%**<br>This segment has a low motivation and low perception of their ability to eat healthily. They prefer foods that are easy to prepare/most convenient and opt for food that is inexpensive. They frequently consume foods that they grew up with and occasionally consume food because it is trendy. This group was younger (majority 20-24yrs) and less educated (high school education only).<br>**Segment 2: Weight conscious - 51.4%**<br>This segment has a stronger motivation and belief in their ability to eat healthily. They less frequently consume food that is convenient/easy to prepare and rarely consumed food because it was trendy. This group was older (majority 25-29) and were more educated (38% held bachelor degree).

Koksal; 2019; Lebanon	N/R	411; <30-45+ (range); 49.6% male, 50.4% female	Developed their own questionnaire including the food choice motive scale which was previously used in the literature. *Questions under 8 food choice motives (ecological, sensory, convenience and availability, health. weight, mood, price, religion).*	Cluster analysis; 2 step	4	**Segment 1: Careless - 14.3%**<br>This segment had the lowest mean score on each food choice factor and all the attitude measures. They were mostly male (61%), had higher level education (91.5%), aged under 45 (84.8%), and earned more than $2000 a month (66.1%).<br>**Segment 2: Conscious - 35.0%**<br>This segment had the highest score on every food choice factor and all of the attitudes except exercising. This segment was evenly distributed in terms of gender and age, and the majority have a university degree (58.3%) and work less than 20h per week (52.1%).<br>**Segment 3: Hedonic - 20.6%**<br>This segment had the second highest scores on sensory, convenience and availability, and religion dimensions of food choice. They scored third highest on healthy eating and organic/natural food and lowest on food quality and exercising. This segment has more females (56.5%) than males, and is mostly university graduates (60%).<br>**Segment 4: Health and weight conscious - 29.9%**<br>This segment had the second highest scores on ecological, health, weight and price factors in relation to food choice motives. They had the highest scores for exercising and the second highest on the other attitude measures. This segment was almost evenly distributed in terms of gender, mostly under 45 years old (82.9%), and the majority have a university degree (69.1%).

Lara et al; 2014; United Kingdom	N/R	206; 61 (7); 40% male[a], 60% female[a]	Perceived barriers to healthy eating.	Cluster analysis; 2 step	3	**Segment 1[d] - 21.0%**<br>This segment had the largest number of Perceived Barriers to Healthy Eating (PBHE) - e.g. busy lifestyle, irregular working hours, or the belief that healthy eating involves lengthy preparation. They also reported the lowest adherence to the Mediterranean Diet and had the lowest Health Behaviour Score. This segment was younger (mean age 57.2 years) and more people were overweight (mean BMI = 28kg/m^2).<br>**Segment 2[d] - 46.5%**<br>This segment had a lower number of PBHE - they lacked willpower and found it hard to give up liked foods. This segment scored higher in Mediterranean Diet adherence and had a higher Health Behaviour Score. This segment was older on average than people in Segment 1 (mean age 61.6 years).<br>**Segment 3[d] - 32.5%**<br>This segment had the lowest number of PBHE. They scored highest for Mediterranean Diet adherence and had the highest Health Behaviour Score. This segment was the leanest (mean BMI = 25.5 kg/m^2) and the oldest on average (mean age 61.7 years).

Liu et al; 2020; China	Means-end chain theory	438; 69.5 (6.9); 47.7% male, 52.3% female	Developed their own questionnaire. *Questions under 5 factors (food safety beliefs, light product interest, food taste beliefs, food freshness beliefs, general health interest).*	Cluster analysis; 2 step	3	**Segment 1: Health and safety concerned - 38.6%**<br>This segment scored highest for interest in general health and light products and are most concerned about food safety and freshness. They attached the highest importance to goals concerning eating a healthy and safe diet, choosing foods you enjoy and eating in nice surroundings. This group scored lowest for food taste beliefs, level of satisfaction with food-related life and mental health status. There was a weak but positive correlation between satisfaction with life and satisfaction with food-related life. This segment has the highest proportion of females (62.1%) and the lowest level of satisfaction with their economic status.<br>**Segment 2: Hedonic and less health concerned - 29.4%**<br>This segment placed the most importance on the taste and safety of food. They had the lowest scores for food health and assigned the least importance to eating in the company of others, maintaining cultural traditions and controlling weight. They had a middle level of satisfaction with food-related life and self-rated mental health status and the correlation between their SWFL and SWL was the strongest. This segment has the highest proportion of participants without grandchildren living with them (63.8%).<br>**Segment 3: Less safety and somewhat health concerned - 32.0%**<br>This group had the least interest in light products and food safety and freshness. They had the highest level of self-rated mental health status and satisfaction with food-related life and their satisfaction with food-related life was positively and moderately related to their life satisfaction. This group had the highest proportion of males (57.9%).<br>There were no significant differences in terms of age, education, income and living arrangement (with spouse, with children or alone) among the three segments.

Milosevic et al; 2012; Western Balkans	N/R	2813; 45.9; 48.2% male, 51.8% female	Food Choice Questionnaire. *Questions under 8 factors (health and natural content, mood, preparation convenience, purchase convenience, sensory appeal, price, weight control, familiarity and ethical concern).*	Cluster analysis; 2 step	5	**Segment 1: Food enthusiasts - 23.1%**<br>This segment placed a greater emphasis on sensory appeal (eg. tastes, looks and smells nice) and convenience of preparation. They had the highest scores for weight control, familiarity and ethical concern and ranked price as the least important. They have a high consumption of fruit, products with health claims and traditional dishes. They have the highest knowledge of health claims, the most females of all the segments (59.7%), and a mean age of 47.1 years. Of the 6 Balkan countries, Slovenia (13.5%) and Montenegro (16.6%) were under-represented.<br>**Segment 2: Unconcerned food consumers - 22.2%**<br>Segment 2 had a weak overall interest in food and placed the least emphasis on health and natural content. Their scores for weight control, price, sensory appeal, purchase convenience, preparation convenience and mood were below average, and their scores for familiarity and ethical concern were higher than average. They have an average level of fruit consumption, the lowest level of knowledge of products with health claims and level of education. Males (53.9%), rural households (47.3%) and individuals from FYRoM (29.0%) and Montenegro (26.2%) were overrepresented.<br>**Segment 3: Price oriented and distressed - 21.0%**<br>This segment placed the highest emphasis on price and convenience of both purchase and preparation. They scored lowest on sensory appeal, familiarity, ethical concern, and weight control and their fruit consumption was average. This group had the lowest incomes, the gender profile was similar to that of the overall sample (50.5% female), pensioners under-represented (18.3%) and there was a high proportion of households with children (40.8%). The head of the household was significantly younger than the sample average (42.4 years), the prevalence of obesity was low (11.6%) and there was a high number of Serbian households (29.4%).<br>**Segment 4: Purchase convenience - 17.2%**<br>This segment placed the greatest emphasis on purchase convenience and the least significance on familiarity and ethical concern. They had the lowest mean score for price and below average scores for weight control, familiarity and ethical concern. They have a high consumption of traditional meals, the highest income, more males (53.6%) than females,

and more pensioners (27.4%) and Croatians (22.2%). They also had the highest incidence of obesity (15.6%).

Segment 5: Health oriented - 16.5%

This group placed greater emphasis on health and natural content and price was relatively important. The lowest importance was given to mood and sensory appearance. They have a high consumption of fruit and health claim products, have the highest knowledge of health claims and are likely to trade off hedonic pleasure for value and health benefits. This group had more females (55.2%), pensioners (27.8%), the highest level of education and individuals from Slovenia (29.1%). Rural (38.1%) and Serbian households (9.4%) were underrepresented. 9.7% of this segment was obese.

Montero-Vicente et al; 2019; Spain	N/R	500; 25-74 (range); N/R	Food-Related Lifestyle instrument (modified). *Questions under 5 factors (interest in cooking, interest in natural products, quality/price ratio, extra-domestic and social consumption, interested in nutrition and innovation).*	Cluster analysis; Ward's method	4	**Segment 1: Total indifference - 4.0%**<br>This segment is disinterested in nutrition and natural products. There is a degree of indifference towards the value for money and the convenience of products. They consume fruit daily. This segment had the highest proportion 65-75 year olds (45%) and the most households composed of one to two members (50%).<br>**Segment 2: Little time to cook, concerned about nutrition and extra-domestic consumption - 26.4%**<br>This segment is interested in nutrition and health. They show no interest in the price of products, convenience foods or liking cooking, not due to a lack of interest but because they have no time for cooking. Half of this segment consume fresh fruit three times a day or more. This segment had a greater proportion of individuals between 45 and 54 years old (30%) and 41% of households consisted of one to two people.<br>**Segment 3: Cooks and preference for natural products - 40.2%**<br>This segment has an affinity for cooking and a preference for natural products and cooking at home. They show no interest in nutrition and innovation. Price is important to this segment. They have a medium-high consumption of fresh fruits. This segment had a high proportion of four-person households (34.8%).<br>**Segment 4: Unconcerned - 29.4%**<br>This segment is similar to the "Total Indifference" segment, but has a certain interest in cooking and concern for nutrition (however their interest is still lower compared to the other segments). They consume less fresh fruit than the rest of the segments. This segment had the highest proportion of individuals aged under 35 (21.1%).

Naughton et al; 2017; Ireland	Transtheoretical Model of Behaviour Change; Theory of Planned Behaviour	477; 18-65+ (range)[b]; 50.0% male; 50.0% female	Developed their own questionnaire based on scales previously used in the literature. *Questions under 6 social cognitive factors (confectionery consumption, hedonic hunger, perceived behavioural control, dietary planning, perceived need, lifestyle goal).*	Latent class analysis	4	**Segment 1: Triers - 20.0%**<br>This segment was less likely to have decreased sugar consumption (67%) over the past 6 months despite everybody having a sugar-related lifestyle goal. They consumed the most sugar in grams per day despite a low level of perceived need for confectionery. They also had a higher probability for strong hedonic hunger. This group was more likely to be female, aged 18-34 years old, and in the 'at risk' BMI category.<br>**Segment 2: Successful actors - 17.0%**<br>This segment had high probabilities for perceived need for confectionery, perceived behavioural control and dietary planning. The majority (92%) had decreased their sugar consumption over the previous six months and 96% had a sugar-related lifestyle goal. This segment was more likely to have more females than males.<br>**Segment 3: Thrivers - 28.0%**<br>In this segment virtually nobody had strong confectionery consumption or hedonic hunger. This segment was more likely to have more males and people with a healthy BMI.<br>**Segment 4: Unmotivated - 35.0%**<br>This segment had no lifestyle goals and virtually no decrease in confectionery consumption over the past 6 months. They had high probabilities for hedonic hunger and low probabilities for perceived need for confectionery, perceived behavioural control and dietary planning. This segment was most likely to be younger and in the 'at risk' BMI category.

Pentikainen et al; 2018; Finland and Germany	Self-Determination Theory	Finland: 1060; 18-74 (range); 52.3% male, 47.7% female Germany: 1070; 18-74 (range); 49.7% male, 50.3% female	Three-Factor Eating Questionnaire (modified). *Questions under 3 factors (emotional eating, cognitive restraint, uncontrolled eating).*	Cluster analysis; 2 step	4	**Segment 1: Susceptible - Finland (20.4%), Germany (19.8%)** This segment had high scores for uncontrolled and emotional eating and low scores for cognitive restraint. They are susceptible to both external and internal cues for eating and experienced discontent with their eating habits. They experience negative emotions more frequently and make relatively unhealthy food choices (more unhealthy snacks, convenience foods and sweets). This group was younger (majority 18-29 years) and had slightly more female than male respondents (60% female from Finland, 51% from Germany). **Segment 2: Easy going - Finland (30.8%), Germany (29.8%)** This segment has a carefree eating style and are content with their eating habits. They experience negative emotions less frequently than the "Susceptible" or "Struggling" segments. They also consumed less foods belonging to the category of healthy foods than the "Rational" segment. This group was older (majority 50-59 years) and had slightly more male than female respondents (60% male from Finland, 54% from Germany). **Segment 3: Rational - Finland (29.5%), Germany (31.3%)** This group had high attempts to consciously restrict eating, but had low tendencies for emotional and uncontrolled eating. They experience more positive emotions and vitality, are content with their eating habits, consume more healthy foods and less unhealthy snacks and convenience food. The majority of this group was from the 60-74 year age group and there was slight more male than female respondents (57.5% male from Finland, 51% Germany). **Segment 4: Struggling - Finland (19.3%), Germany (19.1%)** This segment had high scores for uncontrolled and emotional eating and cognitive restraint. They had a high susceptibility to external and internal triggers for eating and made strong attempts to control eating. They often consumed unhealthy snacks and convenience food and indicated discontent with their eating habits. The majority of this segment was from the 18-29 age group, Finland had slightly more female respondents (54.4%) and Germany had slightly more male respondents (57.4%).

Rejman et al; 2019; Poland	N/R	600; 18-65+ (range); 38.3% male, 61.7% female	Developed their own questionnaire. *Questions from 14 food choice determinants (e.g. price, taste, nutritional value).*	Cluster analysis; k-means	3	**Segment 1: Non-Adopters - 17.0%**<br>This segment downplayed the importance of sustainability in their food choices and diet-related behaviour. The key determinants of food choice for them (apart from taste) were product quality and price. Low scores were given to seven of the eight determinants that characterize a sustainable diet. This segment had a high proportion of males (61.0%) and people aged between 18-35 years old (35%).<br>**Segment 2: Emergents - 32.0%**<br>In this segment food choices were driven by healthy nutrition, environmental protection and the method of purchased food (cultivation/animal breeding/food processing). They did not consider the full impact of their purchasing behaviour on the natural environment, and believed that their diet is healthy. This segment had a high proportion of females (57.7%) and people aged between 50-65 years old (36.7%).<br>**Segment 3: Adopters - 51.0%**<br>In this segment the top three scoring factors influencing food choice were: food quality, healthy nutrition, and buying local food to support domestic producers. Their food choices were based on the impact on their own health and the natural environment in the highest degree and they expected purchased food products not only to be of good quality, but have a beneficial influence on health and taste good, but also to be produced with low environmental impact. This group was the most likely to be convinced to make sustainable food choices. This segment was mostly female (71.7%) and aged between 50-65 years old (48.7%).

Saba et al; 2019; Italy	N/R	1224; 36.9 (12.8); 39.0% male, 61.0% female	Health and Taste Attitudes Scale (HTAS). *Questions under 3 sub-dimensions of the HTAS (general health interest, light product interest, natural product interest).*	Latent class cluster analysis	3	**Segment 1: Low health interest - 28.2%**<br>This segment placed less importance on health and quality aspects (healthiness, organic foods and freshness) and were the least interested in product information, specialty shops or using a shopping list. They were also less interested in cooking and consumed the highest amount of 'red meat + preserved processed meat products' and saturated fat, and lowest amount of vegetables. This segment had a greater proportion of females (54.2%) and people aged between 18-30 years (53.1%).<br>**Segment 2: Medium health interest - 53.4%**<br>This segment was interested in product information when shopping, had positive attitudes towards specialty shops and used a shopping list when buying food. They placed high importance on quality aspects (food healthiness and freshness). Cooking was regarded as an enjoyable activity. This segment had a greater proportion of females (59.9%).<br>**Segment 3: High health interest - 18.4%**<br>This segment had the highest interest in health, natural products, product information, specialty shops and using a shopping list. They valued healthiness, freshness and had interest in novelty and organic foods. They consumed lower amounts of red meat, saturated fat and alcohol, and higher amounts of fruit, vegetables and fish. This segment was mostly female (75.6%) and had many people aged between 46-60 (48.0%).

Sarmugam et al; 2015; Australia	N/R	530; 49.2 (16.6); 41.7% male, 58.3% female	Developed their own questionnaire based on scales previously used in the literature. *Questions from two scales (impulse buying and food involvement).*	Cluster analysis; 2 step	3	**Segment 1: The impulsive, involved consumers - 33.4%**<br>This segment reported a high level of involvement with all food-related experiences. They had impulsive buying tendencies, particularly during food shopping. They had significantly more frequent consumption of fast foods, take-aways, convenience meals and salted snacks, and the most frequent use of ready-made sauces and mixes. This segment had a higher income than others and had a higher proportion of females and younger adults.<br>**Segment 2: The rational, health conscious consumers - 39.2%**<br>This segment reported the lowest levels of impulse buying tendencies and had higher levels of food involvement in meal preparation and making meals from scratch. They were the most likely to engage in healthier dietary practices and reported significantly lower frequencies of eating takeaways from fast-food restaurants and using convenience or ready-meals. This segment had older consumers than the other segments.<br>**Segment 3: The uninvolved consumers - 27.4%**<br>This segment had low levels of food involvement and low importance attached to healthy eating. They had the lowest frequencies of behaviours associated with meal preparation at home (e.g. cooking meals) and reported the lowest consumption of vegetables. The demographics of this segment were similar to the 'rational, health conscious' segment. Fewer people had high incomes in this segment.

Schnettler et al; 2017; Chile	N/R	372; 20.4 (2.4); 43.5% male, 56.5% female	Developed their own questionnaire based on scales previously used in the literature. *Questions from 3 scales (satisfaction with food-related life scale, food technology neophobia scale, and food neophobia scale).*	Cluster analysis; 2 step	3	**Segment 1: Eating is of little relevance to their families - 24.2%**<br>This segment reported that eating is of little relevance to their families. They had low scores in the three components on the family eating habits questionnaire. They also had the lowest score on the "intangible support" subscale from the family resources scale and lowest scores on the satisfaction with life scale and satisfaction with food-related life scale, although it did not differ statistically from Segment 2. This segment had the highest BMI (mean 26.5), a higher proportion of females (57.8%), and majority were in the middle SES group (34.4%).<br>**Segment 2: Pressured to eat - 25.0%**<br>This segment scored significantly higher than the others in "pressure" - i.e. their family pressured them to eat. They had a low score in "cohesiveness" in the family eating habits questionnaire and low scores for "intangible support" in the family resources scale. They also reported low satisfaction with life and food-related life. This segment had the lowest BMI (mean 22.48), a higher proportion of men (60.2%), and a third were in the low SES group (35.5%).<br>**Segment 3: Enjoy the cohesiveness of family eating - 23.9%**<br>This segment scored significantly higher than the others in "cohesiveness" in the family eating habits questionnaire. They also had the highest scores in "intangible support" in the family resources scale, satisfaction with life scale and satisfaction with food-related life scale although it did not differ statistically from Group 4. This segment had the highest proportion of females (71.9%) and majority were in the middle SES group (43.8%).<br>**Segment 4: Eating is very important to their family - 26.9%**<br>This segment scored significantly higher for the "Importance" part in the family eating habits questionnaire score compared to the other groups. They had the highest score in "tangible support", although it did not differ statistically from groups 1 and 3. This segment had a higher proportion of females (57%) and were in the lower-middle (28%) to middle (28%) SES group.

Schnettler et al; 2017; Chile	N/R	372; 20.4 (2.4); 43.5% male, 56.5% female	Developed their own questionnaire based on scales previously used in the literature. *Questions from 3 scales (satisfaction with food-related life scale, satisfaction with life scale, and food neophobia scale).*	Cluster analysis; 2 step	3	**Segment 1: Neophobic, satisfied with their food-related life - 57.8%** This segment had significantly higher food and technology neophobia scores than the other groups. They also had the highest satisfaction with food-related life, although it did not differ statistically from Group 2. This segment had a greater proportion of students who were not willing to purchase food of plant origin produced with nanotechnology (20.9%), food of plant origin in packaging produced with nanotechnology (15.3%), GM food of animal origin (40.5%), food of plant origin (33.0%) and animal origin produced with cloning (45.1%). This group had a higher proportion of males (59.0%). **Segment 2: Non-neophobic, satisfied with their food-related life - 28.5%** This segment had low scores on the food neophobia scales. There was a greater proportion of students who were "very willing" or "extremely willing" to purchase food of plant origin produced with nanotechnology (8.5 and 6.6%, respectively), food of plant origin in packaging produced with nanotechnology (19.8 and 9.4%, respectively) and GM food of plant origin (both 10.4%). This group had a greater proportion of females (54.7%). **Segment 3: Food neophobic, unsatisfied with their food-related life - 13.7%** This segment had higher food neophobia than Group 2 and the lowest satisfaction with food-related life. This group had a higher proportion of males (66.7%). The authors decided not to discuss in detail the results referring to Group 3, due to the risks involving drawing conclusions based on a segment with a low number of consumers (n=51).

Schnettler Morales et al; 2016; Chille	N/R	372; 20.4 (2.4); 43.5% male, 56.5% female	The Family Eating Habits Questionnaire (FEHQ). *Questions from 3 components (importance of eating to family members, cohesiveness of family eating, pressure to eat).*	Cluster analysis; 2 step	3	**Segment 1: Neophobics satisfied with their life and their food-related life - 26.9%** This segment presented the highest score on the food neophobia scale, (although it did not differ statistically from Group 2). The scores obtained on the satisfaction with life scale and satisfaction with food-related life were similar to Group 3 and significantly higher than Group 2. This segment contained a greater proportion of students who perceived their health as very good (49%). This group was majority non-Mapuche origin (92%). **Segment 2: Neophobics moderately satisfied with their life and their food-related life - 40.8%** This segment had a score on the food neophobia scale that was statistically similar to Group 1. Presented the lowest scores on the satisfaction with life scale and satisfaction with food-related life. They reported the highest number of days affected by mental health problems and contained a significant proportion of students who perceived their health as fair (25%).This group had the highest proportion of people of Mapuche origin (19.7%). **Segment 3: Non-neophobics satisfied with their life and their food-related life - 32.3%** This group had the lowest score on the food neophobia scale. The scores obtained on the satisfaction with life scale and satisfaction with food-related life were similar to Group 1 and there was a greater presence of students who perceived their health as excellent (15.8%). This group was majority non-Mapuche origin (85.8%). *There were no significant differences for gender or age in any of the segments.

Simunaniemi et al; 2013; Sweden	N/R	1191; 18-64 (range)[b]; 56.0% male[a], 44.0% female[a]	Developed their own questionnaire based on scales previously used in the literature. *Questions from 5 factors (determinants of fruit and vegetable consumption, habit, perceived barriers, perceived physical environment, knowledge).*	Cluster analysis; 2 step	2	**Segment 1: Positive cluster - 40.0%** This segment was generally favourable towards fruit and vegetables. Those most likely to be in the positive cluster were those looking for information regarding diet and health. Barriers to healthy eating were not liking fruit and vegetables or having health problems. This segment was older (mean age 54.3 years), had a higher proportion of women than the indifferent cluster (63.2%), and consumed more fruit and vegetables. **Segment 2: Indifferent cluster - 60.0%** This segment had more neutral perceptions regarding fruit and vegetables. Those most likely to be in the indifferent cluster agreed that fruit and vegetables are difficult to store at home and that preparation takes too much time. Barriers to healthy eating were not liking or not having fruit and vegetables as a habit, being lazy or forgetting, and a lack of time. This segment was younger (mean age 46.6 years) and had more men than the positive cluster (48.1% male).
Voinea et al; 2019; Romania	N/R	1185; 18-65+ (range); 35.7% male, 64.3% female	Developed their own questionnaire. *Questions under 5 factors (interest in a healthy diet, importance of taste and other sensory characteristics in choosing consumed foods, importance of the nutritional value of the diet, the degree of blog use that contain topics about healthy eating, and BMI).*	Cluster analysis; 2 step	2	**Segment 1: Interested - 57.5%** This segment consisted of consumers with a high level of interest in healthy food. They are interested in the nutritive value of products they select and have a balanced diet. Their consumers' concern for a healthy diet is correlated with their proneness for using social media and specialized blogs in order to search for specific information on healthy food. The mean BMI of this segment was in the normal weight range (22.6kg/m^2), the majority of people were female (74%), and this segment was slightly younger than segment 2. **Segment 2: Eclectics - 42.5%** This segment had a lower interest in healthy food and a moderate interest in healthy eating. Consumers lacked well-founded principles of healthy eating, and may prioritise immediate pleasure and their desires rather than following a consistent food consumption pattern. Give higher importance to taste and other sensory characteristics of products, and lower importance to the nutritional value of their diet. This segment had low usage of specialized health blogs. The mean BMI of this segment was overweight (25.1kg/m^2) and there was an equal amount of male and females.

Wetherill et al; 2018; USA	Hierarchy of Food Needs Model	73; 41.5 (14.9)[b]; 27.2% male[a], 72.8% female[a]	Food Choice Values Questionnaire (modified). *Questions under 8 factors (convenience, accessibility, tradition, comfort, organic, safety, sensory appeal, weight control/health).*	Cluster analysis; 2 step	4	**Segment 1: Limited endorsement of food choice values - 23.0%** This segment was the least likely to strongly endorse any of the eight food choice values. Only two of the eight food choice values were moderately endorsed: sensory and accessibility. This cluster also placed a lower priority on food choice value subscales related to safety, health/weight control, organic, tradition, and comfort. This cluster was the youngest (mean age of 32.8) and nearly half were unemployed (47.1%). **Segment 2: Safety and sensory - 33.0%** This segment strongly endorsed several food choice values, including safety and sensory appeal. While not as extreme as the first cluster, this group also placed a lower priority on the food choice value subscales related to health/weight control, organic, tradition, and comfort. This segment had a wider age range (20–75 years) than the others and over one-third of members were unemployed (39.1%). **Segment 3: Health and weight control - 18.0%** This segment placed a higher priority on health and weight control, which was also the food choice value most strongly endorsed of all eight; however, this cluster overall did not demonstrate a strong endorsement for any of the food choice value subscales. The mean age of this segment was 37.8 years and over two-thirds of the members were employed (69.2%). **Segment 4: Broad endorsement of many food choice values - 26.0%** This segment had strong endorsement of multiple food choice values subscales, including safety, sensory, accessibility, convenience, and health/weight control. Additionally, this cluster demonstrated moderate-level endorsement of organic and traditional values as factors that influence their daily food choices. This segment was the oldest with a mean age of 48.0, an age range between 24 and 75 years, and 94.4% were employed.

FOOTNOTES:

BMI: Body Mass Index, SES: socio economic status, FYRoM: Former Yugoslav Republic of Macedonia. N/R: not reported S1: Study 1, S2: Study 2

[a]studies that reported sex – not gender

[b]demographics (i.e. age & gender/sex) reported for the total sample rather than the number of people involved in segmentation

[c]gender/sex is not differentiated so classified as N/R.

[d]segments did not have names
[e]percentages reported as in the paper and do not add up to 100%
[f]demographics not reported for individual segments
